# What constitutes an employer of choice? A qualitative triangulation investigation

**DOI:** 10.1186/s12960-024-00928-7

**Published:** 2024-06-18

**Authors:** Mohamed Mohiya

**Affiliations:** Human Resources Management Department, College of Business, King Khalid University, Abha, Saudi Arabia

**Keywords:** Employer of choice, Social exchange theory, Thematic analysis, Triangulation, Saudi Arabia, Qualitative

## Abstract

Employer of choice (EOC) is a relatively new phenomenon, particularly in Human Resources Management. Existing employees and prospective talent have reasons and expectations to designate an employer as an EOC. While EOC has received extensive attention from both academics and practitioners over the past few years, the work has mostly focused on managerial and marketing perspectives, and thus far lacks a strong theoretical foundation. Drawing on Social Exchange Theory (SET), based on Human Resources and employees’ perceptions and experiences, this research aims to explore and investigate the factors that constitute/designate an employer as an Employer of Choice EOC. Two qualitative triangulated data sets were collected from existing full-time employees at a Saudi multinational corporation: open interviews and document analysis (cross-sectional and longitudinal). Thematic analysis (TA) was employed to analyze both methods. The findings reveal that company image, training, and development, satisfaction, involvement and commitment, fairness, work culture, reward, opportunities for growth, teamwork, motivation, and corporate social responsibility are the factors that lead employees to designate an employer as an EOC. This research contributes to knowledge conceptually, theoretically, and empirically, mainly in the area of Human Resources Management. This research represents one of the first studies to empirically identify and investigate employee-related factors and evaluate them all together in a multinational Saudi organization. Recognizing the findings of this empirical-based research assists HR managers in designating their organizations as an EOC for current employees and prospective talents.

## Introduction and research background

One of the top priority goals that strategic HR focusing is to make their organizations designated as Employer of Choice (EOC) to attract and retain talents. In the past few years, companies around the globe have experienced some competition in attracting talented employees [58]. Companies, therefore, utilise their resources to become an employer of choice [47]. The war for talent has become one of the top issues for strategic human resources [67]. One strategy that is likely to become a winner in this talent competition is inducing employees to designate an employer as an Employer of Choice (EOC) [50, 59].

The very existence of the concept of an EOC suggests that employees deliberately choose to work for an EOC instead of for other companies [20]. However, as a concept, Employer of Choice (EOC) is still a relatively new phenomenon, particularly in Human Resources. Based on an analysis of the literature, there are other similar concepts, such as employer branding. Ambler and Barrow [1], who coined the term “employer brand”, conceptualized it *“as the package of functional, economic and psychological benefits provided by employment, and identified with the employing company”*(p. xvi). Backhaus and Tikoo [4] defined employer branding as “a targeted, long-term strategy to manage awareness and perceptions of employees, potential employees, and related stakeholders with regards to a particular organization” (p. 2). Employer branding has further been conceptualized as “a targeted, long-term strategy to manage the awareness and perceptions of employees, potential employees, and related stakeholders with regards to the particular firm” (Sullivan, 2004: 1). In addition, employer branding has been conceptualized as “as building an image of an organization to distinct and desirable employers” ([24], 48). Nevertheless, like many others, these conceptions in the stream of research on the employer of choice have focused on organizational and managerial perspectives to achieve organizations’ strategic goals. Most importantly, the research has clearly neglected employees’ issues. The present research defines Employer of Choice as the needs and expectations that attract employees to designate an employer as an Employer of Choice.

From the employees’ perspective, it can be considered that an EOC is a place where they are interested in or enthusiastic about working while existing employees are interested in continuing in that workplace and are content with the facilities available. According to Armstrong [2], employer branding creates EOCs for individuals and instills in them the desire to continue with a given employer. In a different dimension, an employer of choice is summed up by the popular phrase “a great place to work”.

From organizations’ perspectives, there is increasing competitiveness in the job market and the race for talent has generated a requirement on the side of the employers to prove themselves worthy by engaging in different strategies to retain and attract potentially talented people. It has become necessary for employers to attract and retain competent and enthusiastic employees so that all stakeholders are satisfied and the organization is capable of contributing towards business success. Numerous mechanisms are adopted by employers to transform themselves into Employers of Choice (EOC). Nevertheless, even if a firm makes a great effort, no guarantee existing and future employees will consider that company to be their employer of choice. However, a small portion of job seekers consider the status or brand of the employer while deciding to choose or associate with an employer [26].

There are various attributes regarding EOCs where employees play a critical role in designating an employer as an EOC. Some of these include competitive pay and benefits, the provision of a reasonable degree of security, quality of work life, enhanced future employability, commitment, employer image, supportive leadership, participation of employees, psychological benefits, opportunities for growth, and learning and recognition [2, 27, 28, 31, 32, 46] [52, 70]. An EOC provides an incredible work atmosphere, culture, climate, and workplace environment to attract and retain a highly competent workforce. The characteristics of an EOC may aid both the workforce and customers in terms of holistic well-being. Large numbers of progressive organizations have set themselves the goal of becoming an EOC, where people are willing to work at any cost, not only for financial benefits but also for psychological and functional benefits. Thus, the assessment of an employer as an EOC involves working with an exceptional employer who recognizes the achievements of employees in the workplace. Noe [33] proposed that an employer can be successful through a rigorous evaluation process of determining the leadership qualities, best practices, and culture that would be assets to attract and manage the most talented employees in achieving their goals.

Theoretically, due to a lack or absence of a strong theoretical foundation in EOC research, mainly social theory, this research adopts Social Exchange Theory (SET) for several reasons. First, SET is one of the most influential theories in business and HR mainly found useful in explaining the relationship between employees and employers which is based on reciprocity conveying benefited resources [15, 16, 38]. The approach has the distinct advantage of recognizing employees’ interpersonal and social issues. Second, SET is relational to the context and aim of the present research. Third, this current research is a qualitative study driven by social theory that has been adapted in advance of the data collection. The role of theory is fundamental as a vehicle in the present research. However, qualitative scholars often use theory as something that emerges from the data collection and analysis [12]. Fourth, the theoretical lens of SET assists in serving the main aim of this research by offering a clearer explanation and better understanding to identify and investigate this new phenomenon, EOC, and factors that designate their EOC. SET sees the factors that contribute to EOC as resources. In general, the relationship between reciprocity and resources in SET is interdependent. Employers need to provide employees with resources that will oblige them to reciprocate in kind with engagement [38]. In other words, there is no reciprocity without resources. Ultimately, reciprocity within EOC contains and conveys resources. Employees will choose to produce in response to the resources they receive from their employer of choice [38]. According to Cropanzano and Mitchell [15], once employees receive socioemotional and economic resources from their employer, they, in return, feel obliged to respond in kind and repay the employer. Therefore, the resources/factors of SET assist in investigating the types of resources that employees expect to receive from employers.

Methodologically speaking, most existing business and HRM studies about EOC in relation to marketing only use wither single quantitative method which indicates there is a qualitative methodological gap, particularly triangulation methods in HR studies. Within the context of the current research, a qualitative approach is not only appropriate but also needed. The two qualitative methods help to uncover unknown antecedents that contribute to designating the employer as an Employer of Choice in a new and undiscovered context, Saudi Arabia organization. Based on the evaluation of relevant empirical studies, the researcher realizes that the approach drawn from the research questions and the overall strategy of the research required a need for qualitative triangulation research methods, compared to a quantitative method. The advantage of qualitative research is that it allows the researcher to gain a greater perspective into the insights of the participant because it provides the opportunity for the power of words to prevail. An example is a semi-structured interview. Instead of tick boxes and Likert scales (quantitative research), qualitative research asks for self-expression and an interpretation of how the subject feels and understands. A qualitative approach seeks answers to questions that stress what and how social experience is created and given meaning. In contrast, quantitative studies emphasize the measurement and analysis of causal relationships between variables, not processes.

The two triangulated qualitative methods that will be used in the present study are semi-structured interviews and a document analysis approach (combined longitudinal and cross-sectional designs). The two qualitative methods are ‘equally and parallel’ which can be viewed as exact equivalents to serve the purpose of the study by addressing the research question. Most importantly, these two triangulated methods will help improve objectivity on the limitations of qualitative methodology is low objectivity. Moreover, the two qualitative sources offer rich data to answer the research questions sufficiently. In addition, using triangulation methods will minimize the common method bias.

The two approaches tend to be available for data collection in research studies: longitudinal and cross-sectional research—this research uses both. The present research, through having data from the document that provides reactions accumulative of employees’ experiences about EOC covered 2 years long, typically fits the description of longitudinal research. For example, the document analysis covers 2 years and the semi-structured interview covers 3 months. For the present research, both cross-sectional and longitudinal provide rich accounts of the employees’ accumulative experience.

Contextually, the demands and needs for EOC differ from country to country due to business, social, and cultural differences. Based on the analysis of the relevant literature about EOC in the Middle East, particularly Saudi Arabia, it found limited empirical-based evidence studies.

Contextually, based on a review of the literature, it appears there is a lack of empirical knowledge concerning the factors that contribute to EOC designation, especially with regard to employees’ perspectives in the Middle East, particularly in Saudi Arabia. This present research seeks to address this contextual knowledge gap. This study aims to identify and investigate relevant employee-related factors. The study assumes significance since no such attempt has yet been made concerning EOCs in the Kingdom of Saudi Arabia. The objective of the study is thus to identify the factors of an employer that contribute towards making it an EOC based on Social Exchange Theory (SET).

## Social exchange theory (SET) and Employer of Choice (EOC)

Some EOC studies have adopted psychological contract theory and signalling theory (e.g., [37]). Even though Saini and Jawahar’s [37] study focused heavily on the managerial and psychological aspects, these theories did not consider social and employee perspectives.

This research adopts Social Exchange Theory (SET) as a theoretical lens mainly because it magnifies the importance of reciprocity, or two-way processes [15, 16, 38]. There are several reasons for the value of the social theoretical foundation in the present research. First, SET is one of the most significant conceptual approaches in human resources management and organisational behavior and is based on reciprocity between employees and employers in the workplace [15, 16, 38]. Second, SET is also useful in explaining the core conceptualization of the present research—the notion of the employer of choice. In theory, SET recognizes employees as a party that is reciprocally interdependent with employers. The SET mainly determines the relationship between parties involved, i.e., employer and employee, who always maintain a reciprocal interconnected affiliation. Third, and most importantly, SET’s resources are considered as factors that employees need or expect in order to reciprocate and designate an employer as an Employer of Choice. Fourth, Blau [6] suggests that social exchanges are voluntary actions that, in the context of the present research, align with the word “choice”. For example, in the context of the present study, if the employer provides resources to employees, in return, employees are expected to reciprocate that by choosing the employer.

Unlike psychological contract theory and signaling theory [37], SET is a social science theory that considers non-psychological or economic resources in social relationships based on voluntary interactions, not economic transactions. This viewpoint is aligned with other social exchange theorists who suggest, in comparison to economic exchange, that relationships depend on willful actions in contrast to formal actions [3, 6]. Relationships based upon social exchange generally have more intangible resources and focus more on resources related to socio-emotional factors, e.g., cognizance, appreciation, or praise [36]. These intangible resources of SET offer a clearer explanation of how employees view their relationships with employers in the workplace, based on reciprocation and more than mere economic resources.

The present research is driven by SET to explore and identify employee-related factors/resources that designate an employer as an EOC. The role of theory is fundamental as a vehicle in the present research. However, qualitative scholars often use theory as it allows factors to emerge from the data analysis [12]. Conversely, Silverman [43] argued that most contemporary qualitative scholars have become increasingly interested in testing and exploring theories. Undoubtedly, there is no reason to prevent the use of qualitative triangulation research in the testing of theories that have been specified in advance of collecting the data [12]. Further, SET is an ideal theory that could assist in meeting the main aim of the present research of identifying and investigating the factors that make employees choose their employer. SET stipulates that the relationship between reciprocity and resources is interdependent. Employers need to provide employees with resources that will oblige them to reciprocate in kind with engagement [38]. In other words, there is no reciprocity without resources. Ultimately, reciprocity contains and conveys resources. Therefore, a certain amount of various resources is essential for the existence of an EOC. Employees will choose to engage themselves in response to the resources they receive from their employer [38]. According to Cropanzano and Mitchell [15], once employees receive resources associated with their socio-emotional and economic needs from their employer, they, in return, feel indebted and reciprocate with the employer in multiple ways. Therefore, the resources/factors identified in SET assist in investigating the types of resources that employees expect to receive from employers. The ongoing empirical examinations in organizational behavior and development were also taken into consideration to ascertain a fair idea of the concept of EOC and its related factors.

## Relevant EOC empirical work

EOC can be best understood through employer branding, supportive leadership, fairness in recruitment processes, opportunities for growth and development, and retaining and attracting talented employees. Chhabra and Mishra [13] asserted that employer branding reflects the employer’s image and employer-of-choice status, and suggested that the best methods, tools, and techniques must be applied by the employer to motivate, influence, retain, and engage employees. Vinoth and Vasantha [46] conducted a study using a sample of 364 final-year students to examine the utility of employer branding in choosing an employer. They found that psychological benefits offered by a company are more important than other benefits such as financial or economic and functional benefits when choosing the right employer. Jobseekers are likely to be attracted to those firms that exhibit unlimited employer image value in contrast to those who show a low degree of employer value related to the image. However, other factors have not yet been identified, particularly in the Kingdom of Saudi Arabia (KSA), an issue that the present study seeks to address.

Saini and Jawahar [37] studied the influence of employment experience and employer rankings on employee recommendation as an EOC. They also probed whether these variables have an impact on employee characteristics. The study was conducted on 39,010 employees, which took 3-year employer rankings (2015–2017) and revealed that employee recommendations are influenced by employees’ experience in the workplace. Further, they (ibid.) observed that employee characteristics such as full-time vs. part-time, tenure, employment status, and employment experience also influenced employee recommendations pertaining to the company as an employer of choice. However, unlike the present research, Saini and Jawahar [37] focused mostly on managerial perspectives.

In addition, Mau [27] conducted a recent study focusing on determining the notion of branding the public sector as EOC to recruit and retain the leadership ability of people in the service. This study was undertaken to address a challenge encountered by the government in the recruitment of candidates with optimal capabilities for public services. The Canadian Federal Government undertook an initiative in 2007 to brand their public service. The findings suggested that it was very challenging to provide an exact concept of branding for the public sector, where a diversified workforce was employed [27]. Although branding was found to be one of the most popular concepts in the public sector as an EOC, it was found that these concepts had flipsides that required immediate attention. Though the Canadian Federal Government took great pains to develop the concept of branding in the public service, they failed to lead federal public services to be considered as an EOC.

Recently, Tanwar and Kumar [45] conducted a study of college students to ascertain the association between brand dimensions of employers and EOC status. Factor analysis and structural equation modeling were used in the study. Tanwar and Kumar [45] found that person-organisation fit was perceived as a mediator for EOC and that the dimension related to employer brand required a link with person-organisation fit. It was also determined that social media plays a key moderating role in facilitating EOC. Unlike Tanwar and Kumar’s [45] research, the present study adopts qualitative and longitudinal methods that offer in-depth understanding in different ways based on employees’ experiences. Most importantly, unlike the present research, the pieces of research discussed appear are not based on theoretical foundations, which means they can be considered more as practical research rather than scholarly/academic work.

Based on the critical evaluation of the relevant literature, most works have been focused on managerial and organisational perspectives and have neglected employees’ perspectives. This research, grounded in employees’ experiences, addresses this significant gap in the literature. Unlike other managerial and organizational studies, this research, through the theoretical lens of Social Exchange Theory, identifies and explores employee-related factors that attract employees and encourage them to designate an employer as an Employer of Choice. Based on these points, the following exploratory research question was developed.

*What factors attract employees to reciprocate their designation of an employer as an Employer of Choice (EOC)?*

## Qualitative triangulation methodology

The primary notion of qualitative research is to develop an understanding of a point rather than to verify it. Due to this, the outcomes of a qualitative investigation can be considered to be novel, reliable, genuine, and trustworthy, in contrast to quantitative research [19, 25]. However, in qualitative methodology, subjectivity is a matter of concern [11].

With quantitative research, the findings have a higher validity as a result of the high degree of representation [51]—a concern for qualitative research. However, this research uses two triangulation methods, which provide rich data and a consequent increase in validity. For example, in the present study, document analysis, along with the open interviews, are utilized equally to shore up validity.

There is a qualitative methodological gap in the relevant literature about Employer of Choice (EOC). Reflecting on the research question above that emerged from these knowledge gaps, the answers to the research question could be obtained through both qualitative and/or quantitative methods. However, as mentioned in the review section, from the analysis of the relevant studies (e.g. [37]), it appears that a quantitative approach is favored. Therefore, this research addresses this methodological gap by using a qualitative approach. Within the context of the current research, a qualitative approach is not only appropriate but also needed.

To approach the research question, a mixed triangulation of the qualitative approach to uncover unknown factors that encourage employees to designate an employer as an Employer of Choice (EOC). The two triangulated qualitative methods used in the present study are open interviews and document analyses. The two qualitative methods are applied “equally and in parallel” and can be viewed as exact equivalents to serve the purpose of the study by addressing the research questions. To the researcher’s knowledge, this is the first study of EOC that adopts qualitative triangulation methods—in particular interviews and documentary analysis. The obtained document method is a complete set that draws upon first-hand employee comments spanning a 4-year period which is extracted from the internal organizations’ HR Blog system. The total number of comments is 104.

The second method is open interviews conducted with 22 employees. Triangulation methods assist in capturing different dimensions of the same phenomenon. For example, interviews and document analysis support the interrogation of the data to identify and/or explain factors, problems, or causes that affect employees’ decisions to choose an employer. Thus, the need to use triangulation of multiple data sources is crucial not only because it offers richer data, but also because it allows digging in-depth to obtain fine-grained results that capture what is happening in reality.

In general, triangulation is used as a means of cross-examining results from one form of data collection with those of another. For example, the document analyzed in this research contains 104 employee comments over 4 years, and interviews covering 3 months. For this research, the data triangulation helps create greater confidence in the overall results [53]. The two triangulated qualitative methods decrease researcher bias [56]. Multiple qualitative methods are employed in the collecting of data as a means of minimizing bias and limitations inherent in each method ([56]. For example, unlike with open interviews, the document analysis method used in the current research contains 104 comments written first-hand by employees with no involvement from the researcher, which decreases bias.

Two broad approaches are available for data collection in research studies: longitudinal and cross-sectional research. This research draws on both approaches. Because the data from the document provides cumulative employee reactions and employee perceptions over 4 years, this study most typically fits the description of longitudinal research.

The obtained document from the employer was as a complete set which was extracted from the internal HR Blog platform of a large multinational energy corporation. The obtained document contained interactions and discussions between employees about the Employer of Choice subject. This document contains first-hand, unadulterated comments made by employees on the platform. All documents were extracted from the HR Blog as it is without modifications or editing, as the organization stated. These texts, taken from the platform, are directly and purposively relevant to the aim of the present research.

In general, researchers need to analyze the significance of documents about study problems and aims [7]. The sampling characteristic for this research is a purposive sampling technique which is widespread in qualitative research. As this research aims to identify and investigate the factors that constitute/designate an employer as an Employer of Choice (EOC), purposive sampling was used for this study to only focus on full-time employees. For example, in interviews, the purposive sampling technique was used to focus on full-time employees. For the second method, document analysis, the received document contains employees' computerized first hand-typed written where employees responded to a question about “What designated employer of choice?” This topic document was rich and detailed information about employees’ cumulative experiences over 4 years.

For interviews, respondents were enrolled via an email sent by HR inviting them to participate. The email was purposefully sent to all employees working full-time in the organization to increase the chance of diversification of participants’ demographic characteristics. The email contains a brief invitation paragraph and several attachments, namely: a plain language statement (including the author’s contact details), and the interview guide. The researcher was copied in the email and at the end of the email, the HR asked prospective participants to contact the author directly for any questions about the research and, most importantly, to arrange the interview time and location, if they have an interest. The reason behind sending these information sheets all together in advance is to give employees time to read and understand and provide them with a clear idea about the project and interview, as well as give them time to read and decide if they would like to participate. In addition, the researcher also provided each participant with a hard copy of these sheets to explain it to them before starting the interviews.

The organization which the data was collected from is a large multinational energy Saudi corporation located in Saudi Arabia. The participants are full-time employees and the demographic characteristics are high (please see Table 1).

**Table 1:** Participants' demographics characteristics

Demographics	Detailed information	Percentage (%)
Gender	Male	57
	Female	43
Years of experience	1–4 years	37
	5–10 years	22
	11–15	14
	16–25	21
	26–40	6
Positions’ hierarchy	Entry level	44
	Supervisory level	32
	Managerial level	17
	Leadership level	7
Saudis and expatriates	Saudis	54
	Expatriates	46
Departments/functions	Administrative jobs (e.g., HR-finance-marketing)	61
	Technical jobs (e.g., engineers and technologists)	39
Education level	Ph.D./Doctorate	0
	Masters	21
	Bachelors	47
	Diploma	32

Three reasons for settling on only 22 interviews. First, from interview number fifteen and onwards, most of the interviewees’ answers started becoming repetitive. Second, the confirmation and validation between the two methods reached a satisfactory level. For example, as interviews and documents were used equally weighted and parallel, some factors that emerged from the preliminary analysis of the document required further questioning, clarification, or confirmation from interviewees during interviews (and vice versa). Third, the diversity of demographic characteristics of participants was high in genders, types of jobs (technical and administrative), years of experience, nationalities, levels of education background, and position levels (please see Table 1).

For the present research, thematic analysis was undertaken for both methods because it offers some flexibility when analyzing qualitative data. Thematic analysis should be seen as a foundational method for qualitative analysis [10]. Qualitative thematic analysis is a commonly used approach to analyze textual material obtained from a range of sources, including interviews and documents. As defined by Braun and Clarke [10] “*thematic analysis is a method for identifying, analyzing, and reporting patterns (themes) within data”* (p.6)*.* However, for thematic analysis, there is no fixed universal method. While key themes/factors have already been identified as concepts from the analysis of literature, other themes are allowed to emerge and they are coded based on the theoretical lens of SET.

For the present research, the process of analyzing the qualitative data involved: preparation of data; familiarisation with data; generating initial codes; collating similar codes into pre-existing or emerging themes; re-reading and reviewing themes that related to the research questions; and refining themes. This process was done through creative engagement with the data and following intuition [10].

For the present research, the coding process was carried out manually. Unlike other electronic software, Wicks [71] suggests that manual coding provides the researcher with an opportunity to reflect on the analysis as they immerse themselves in the data. However, one of the disadvantages of using manual coding, in particular with large data sets, is that it is less efficient or manageable [40]. As a result, this may lead to missing important aspects of the data. However, for the present research, the author has spent a large amount of effort and time to organize, read, and understand the data ensuring there are no missing key information or relevant factors.

Manually, the analysis of interviews’ transcriptions and documents was completed through the use of thematic analysis by starting with coding key factors that were identified based on the frequencies (presented in the conceptual model). Through the identified themes, the data will be allowed to capture an explanation of possible reality through evidence, which ultimately helps address the research questions sufficiently, as suggested by Braun and Clarke [10]. In the second stage of coding, there were new factors started to emerge based on the data analysis of pre-determined factors. These new emergent factors were coded based on the frequency and relevance of patterns. Through the coding process of thematic analysis, the entire data set is used to explore meaningful, frequent, and relevant patterns that emerge [54].

The use of two different sources of qualitative data has significantly reduced any potential risks of common method variance (CMV) [8]. This present research uses two mixed qualitative methods. Two procedural actions were taken to reduce CMV. First, the data were collected from interviews and documents at two different and separate times. Second, during the coding and thematic analysis stage, some of the key factors emerged from the interviews’ transcriptions and others were allowed to emerge from document analysis, but further confirmation and validation were conducted with other sources/methods to avoid any risks of common method variance. Therefore, the results of the investigated factors revealed that the issue of common method variance was not a major issue in this study.

The use of two mixed methods has assisted in overcoming any risks of bias, e.g., social desirability bias (SDB). First, all participants' personal information in the HR Blog where the documents were extracted from was completely anonymous which reduced social desirability bias (SDB). Second, the document analysis method used in the current research contains 104 comments written first-hand by employees with no involvement by the researcher, which consequently, decreases the bias. Third, for the interviews, in the plain language sheet, I mentioned that all of their responses would be confidential their participation is voluntary and they could leave at any time during the interview. Therefore, the use of two mixed methods has not only helped to decrease SDB and increase the genuineness of responses but also significantly increased the results’ confirmation and validation.

Most importantly, as the present research is theory-driven, the SET lens played a fundamental role in the analysis of the data. The coding techniques of thematic analysis necessarily depend on whether or not the themes are “theory-driven” (Braun and Clarke 2006). In the present research, themes have been analyzed, identified and interpreted, and driven or guided by “resources”, as provided in SET.

## Findings and discussion

The purpose of this discussion section is to theoretically and empirically analyze, interpret, and establish the significance of the findings in the relevant literature, in particular about the research problem being investigated.

The overall theoretical analysis and interpretation of the present study’s results confirm that designating an employer as an Employer of Choice is based on reciprocity between employee and employer in exchanging resources. This is in line with the SET [15, 38] which postulates that employees are involved in a social exchange relationship when they act in favor of another party, with the expectation that this favor is reciprocated in the future. Saks [38] suggested that employees are more willing to reciprocate or exchange their engagement for resources provided by their employer. Moreover, this is consistent with other studies that have suggested that EOC factors in organizations’ context in the workplace depend on reciprocal interactions [9, 20, 35].

Based on the thematic analysis of findings in this study, several factors were identified as significantly affecting employees’ designation of an employer as an employer of choice. The results that emerged from the analysis are summarised in Table 2 below.

**Table 2:** Rank order, frequency, percentage and supported quotations from data

Group	Key factors	Frequencies	%	Quoted examples from the data (interviews and document analysis)
Organization external	Company image	49	57	“As the new changes against the employees and hence affecting the company’s image as being the employer of choice.” *Interview #2* “I believe an employer of choice demonstrates a great reputation to attract the most skillful employees and to develop the current workforce” *Document’s comment #35*
Employees related	Opportunity for training and development	40	46.51	“Training and Development where employees want to develop skills so they can excel in their jobs on a day to day basis and eventually progress up the career ladder. Employees can access plenty of training and development opportunities in the company.”* Document’s comment #25* “To be an employer of choice, I recommend any company to have the best training and development” *Interview #12*
Workplace environment	Work culture and environment	39	45.34	“Employer of choice that offers work culture and workplace environment that attract and retain qualified employees.” *Document’s comment #81* “Attractive work environment is one of the most important reasons to become an employer of choice.” *Interview #8* “I do believe our company is an employer of choice because it has a very positive working environment” *Interview #3* "Being an employer of choice means that the company has carefully thought about creating an environment where people want to work and have long-lasting careers.” *Document’s comment #27* “What makes our company the ‘Employer of Choice?’ It really comes down to the values and how it plays out in the workplace. Creating a ‘great culture’, creates a "great workplace", which in turn helps spread the word as the ‘Employer of Choice’” *Interview #19*
Organization external	Attracting and retaining	36	42	“The company must change to be more attractive to new hires, be more supportive to the current talents before they tend to leave and be more flexible an appreciative for experienced employees.” *Interview #22* “Our employer is an employer of choice, but it should not just be an employer of choice for “now” it should maintain and develop more to attract more.” *Document’s comment #33* “Young people may see our Company as an Employer of Choice, and be enthusiastic to join our workforce. However, all the efforts to recruit and select top talent may come to naught, if retention and loyalty is presumed.” *Document’s comment #17* “To be an Employer of Choice, we need to be attractive for the young talents” *Interview #10*
Employee related	Satisfaction, involvement and commitment	34	39.53	“Set targets for managers and division heads to achieve a specific satisfaction level in their workplace.” *Interview #1* “To become Employer of Choice, the company should show a commitment” *Document’s comment #75* “At an employer of choice, employees feel as if they have the opportunity to be involved. They can make suggestions, think up new products or service innovations, serve on employee committees to plan events and work processes, and attend appropriate meetings and have input on work processes that affect their jobs.” *Interview #6*
Employee + workplace	Fairness	27	31.39	“Fairness: Perceptions of unfair treatment or a workplace that favors certain individuals over others for unknown, undefined reasons, is an anathema to an employer of choice. Employers need to fairly develop and apply policies, treat employees with the same regard and consideration, and make the workplace guidelines clear and enforceable across the board.” *Document’s comment #55* “I believe our company is still an Employer of Choice. However, the management has still to do a lot to sustain this quality and capability by ensuring fairness in job and benefits to the employee comparing with other best employers” *Interview #4*
Employee related	Reward and recognition	19	22.09	“Reward well – People want to be rewarded in recognition for the contribution that they have made to the organization. They also want to be rewarded at a level which is on or above market rate. The new global package certainly moved in this direction.” *Document’s comment #104* “Recognition: Employers of choice provide feedback to employees about their performance, growth prospects, accomplishments, and areas needing improvement regularly. One of the most powerful forms of employer of choice is employee recognition. At an employer of choice, recognition is regular, targeted to real successes, and used to reinforce positive, desired behavior.”* Document’s comment #97* “I believe the company to still be an Employer of Choice based upon the rewards plan it provides.”* Document’s comment #33* “Instead of a monetary award let employees choose their own prize: for example, dinner in a restaurant, a subscription to a fitness club, a gift certificate.” *Interview #9*
Employee related + Org. External	Opportunities for growth	9	10.5	“Undoubtedly, our company is among the most sought-after employers in Saudi. It provides career growth opportunities for ambitious young people to become their Employer of Choice” *Document’s comment #16* “The company has been an employer of choice for so many years that we started to get accustomed to it. This level brings dangers of falling behind as other companies start eating at the same bite: the employable workforce. Our company is still the Employer of Choice for career growth. To be able to stay competitive as an Employer of Choice on the global employment market, the company needs to open up more opportunities of career growth.”* Interview #7*
Workplace	Team work	9	10.5	“Positive relationships with coworkers: On a larger scale, at an employer of choice, because coworkers like and enjoy working with each other.” *Interview #5* “What makes our company an employer of choice is employees’ relationships. Coworkers like and enjoy working with each other.”* Document’s comment #41*
Employee related	Motivation	9	10.5	“The company should look seriously into the motivation level of employees, especially mid-aged ones, who are considered in their peak production stage and it is time for the company to get the most benefit out of them.” *Interview #14* “The key is motivation whether it is through money, position, exposure, etc. If the employees feel motivated, then their employer will be the employer of choice” *Interview #18*
External Org	Concern for society	2	2.32	“Corporate Social Responsibility: Employees want to work for businesses that are trustworthy, ethical, and socially responsible and have a positive corporate culture where staff are treated fairly and with respect. Develop a Corporate Social Responsibility (CSR) program in your business which sets out your values and principles on how you want to do business in an ethical way.” *Document’s comment #73*

Each of the above factors (present in the table), as identified by the respondents is now discussed in detail.

### Company image

It is evident from the table that company image and reputation were of great importance and were ranked first. The vast majority of employees believe that the company image is a fundamental factor for EOC. It can be considered an extremely important factor that could lead to an organization being designated an EOC. Company image can be understood in terms of employees’ desire to continue in the company for a longer period of time or as long as they can. This result substantiates earlier findings mentioned in the literature review (e.g., [4, 18, 21, 45]).

In contrast to a positive image, a negative image might also lead to negative perceptions of the company’s image [21, 44]. However, Lievens and Slaughter [24] reviewed various articles and pointed out both the positive and negative aspects of company image and emphasized that a positive image of a company influences behavior towards productivity. Applying SET, it can be inferred that employees are attracted to an employer not merely for economic benefits but also for a host of non-economic benefits. Therefore, based on the analysis, company image is a socioemotional factor that was found to contribute strongly towards EOC in this study.

The results about the significance of company image and reputation to designate EOC is broadly consistent with many studies (e.g., [55, 60, 64]. Vast majority of participants believe that the employer’s image and reputation in public through the quality of the products’ brand and services influence the public and, consequently, make employees feel proud of their employer.

### Opportunities for training and development

The analysis of the results shows training and development is one of the most important factors that they need and ultimately influence their decision to designate EOC. Employees interested in acquiring new skills through training. In return, employers need to consider this to become EOC. In light of the relevant literature, this result is also in agreement few studies (e.g., [61, 65]. However, these studies did not fully focus on EOC as a concept but focused on organizational performance. For example, Salah [39] suggests that training and development have an impact that leads to an increase in productivity, quality, and performance. These findings were also supported by Karim et al. [63]. Theoretically, Cropanzano et al. [16] and Cropanzano and Mitchell [15] suggest that the employer–employee relationship can be established through reciprocities. Unlike other studies, this study has thus identified an opportunity for drawing on training and development as a significant factor that is capable of contributing toward perceptions of an EOC.

### Company’s ability to attract and retain employees

Being able to attract talents in the market and most importantly retain them is found one of the most critical factors for employees to designate any employer as an EOC. More specific to the context of the organization as an employer, organizational attractiveness refers to the extent to which potential employees view an organization as a desirable and positive place to work [57, 69].

From the table, it can be observed that attracting and retaining talent is one of the vital components of EOC. This indicates that one of the important employer functions is to attract and retain fresh talent with appropriate competencies to achieve organizational success. Participants believe that the talents that the organization attracts will positively influence them. This factor supports other studies that have been highlighted by many researchers [24, 48, 52, 68].

### Satisfaction, involvement, and commitment

One of the key factors found influencing the designation of EOC is employees’ satisfaction, involvement in decision-making, and organizational commitment. Employees believe that these can be achieved via satisfactory compensation and benefits, other amenities, paid holidays, participation in decision-making and job security are factors that could facilitate perception as an EOC. One of the key attributes of SET is that relationships progress over some time with the help of mutual commitment and satisfaction [6, 15, 17, 22, 29, 38]. The findings from this study in this respect align with several earlier studies which suggest that job satisfaction, commitment, and involvement play a key role in making employees feel loyal to the employer [4, 57].

### Fairness

The other significant factor was found to be of importance to employees in developing a positive attitude towards an EOC. Employees perceive that fairness exists in organizations if there are vertical promotions, proper resource allocations, equity, equal treatment, and justice. The current findings from this study are in alignment with previous research by Baldwin [5] and Polayni and Tompa [66]. Molm [30] suggests that fairness is one of the attributes that helps in establishing a good rapport between the employer and employees. Fairness is also found to mitigate conflicts and would be helpful in an employer becoming an EOC. This finding also has its moorings in SET. Treating employees fairly in the workplace mainly in promotion and incentives significantly affects employees’ decision to designate any employer as an EOC.

### Work culture and environment

Considerable evidence exists to support the claim that HR practice and supportive behaviors in the company could create a positive work culture and an outstanding work environment in which employees are interested in working with and continuing to work with the employer. According to SET, employee engagement depends on the nature of the environment and culture provided by the employer to their employees [38]. This is also in line with the findings of Allam [49], according to whom HRM practices help in establishing good working atmospheres or an appropriate culture so that employees consider continuing with the employer.

The analysis of the findings suggests that organizational culture plays a significant role in making an employer an EOC. Outside of EOC’s context, this result is broadly in agreement with several studies (e.g., [62]). It seems that organizational culture is not a minor issue for employees. The analysis and interpretation of the data confirm that the organization's culture becomes a pivotal factor for employees to designate any employer as an EOC.

### Reward

Employers provide appropriate rewards to their employees in return for their commendable performance, which encourages employees to perform further. Rewards refers to offering incentives to employees. Looking at it from the perspective of the SET, as recognized in the literature and the conceptual model, rewards are socioemotional and economic resources that employees may expect to receive from employers. This reciprocity and pattern of exchange is also highlighted in SET [6, 13, 23, 41]. The findings about rewards are supported by the work of Kucherov and Zavyalova [65] and Clark and Oswald [14], who explained that rewards lead to better performances, which could, in turn, lead to the organization being considered an EOC. However, unlike Kucherov and Zavyalova [65] and other managerialists who associate rewards with job performance, the present research focuses on this as a repayment resource for employees to designate an employer as EOC.

### Opportunities for growth, teamwork, and motivation

Career development and growth are found one of the factors that heavily influence the designation of EOC for employees. In the documents, employees pointed out that, at their present company, career prospects are good, employees are part of the growing company worldwide, there is good team conduct, and employees feel motivated when their performance is valued. SET stipulates that decisions made by individuals would be based on expectation of certain outcomes. The factors generated in this study are in alignment with this aspect of SET and alignment with the findings of Cropanzano and Mitchell [15].

### Concern for society

It was found that employees valued their organization’s concern for society. Corporate Social Responsibility (CSR) initiatives aimed at uplifting society have been considered part of leading an employer to be evaluated as an EOC. The interpretation of this finding shows that not just society is affected by CSR, but also employees. It was surprising that employees take the CSR factor to designate an employer's EOC. This found to be significant This finding is consistent with several studies (e.g., [34]). According to Norbit et al. [34], employees tend to have a positive attitude towards the companies that are involved in CSR as it enhances the reputation of the organization among stakeholders. Theoretically speaking, CSR is seen as one of the resources that employees expect from employers to make, in return, an employer as EOC.

**Fig. 1:**
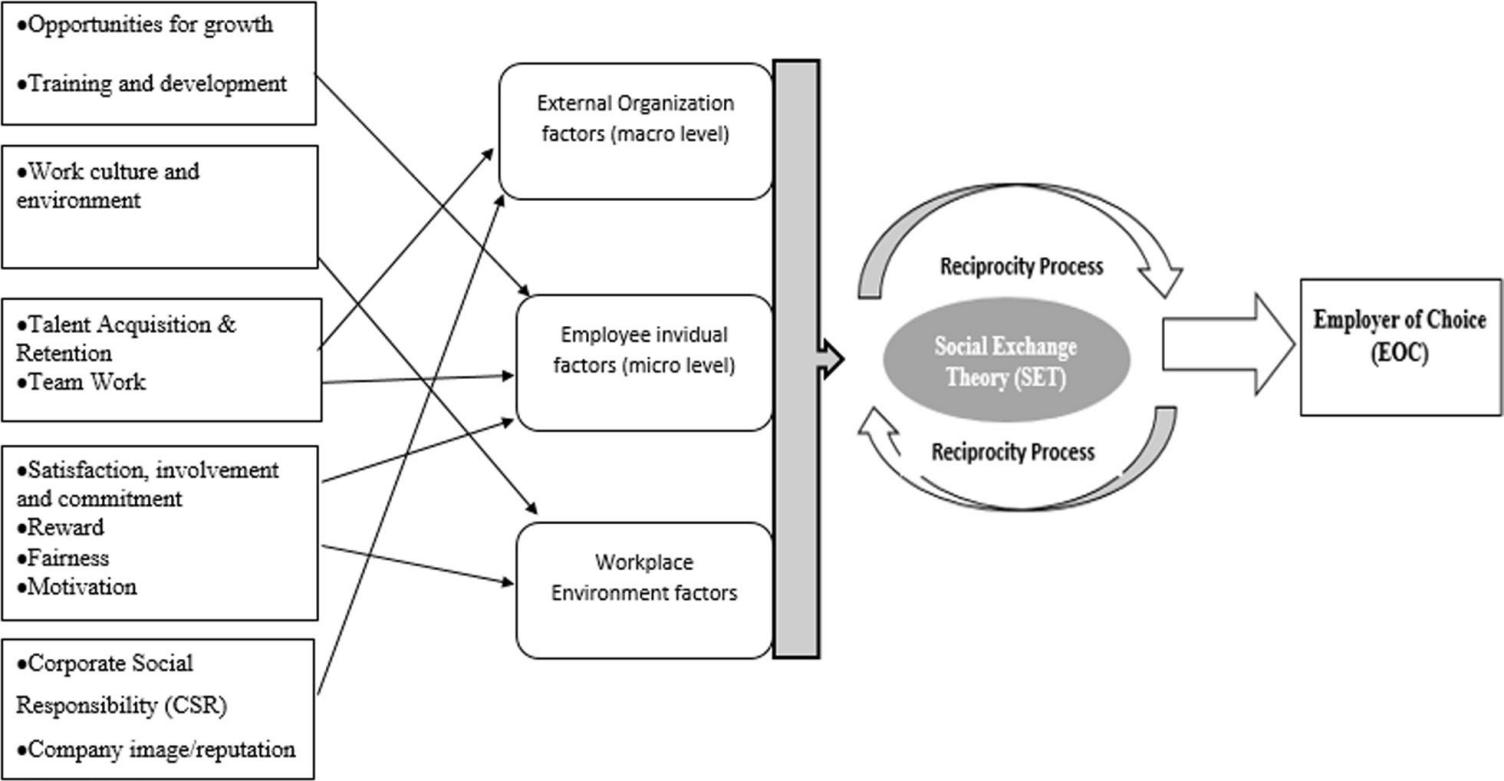
Conceptual model (created by the author)

Based on analysis and interpretations of the findings, through the theoretical lens of Social Exchange Theory, the below figure (Fig. 1) proposes a complex theoretical/conceptual model about the antecedents/factors that encourage employees to designate an employer as an EOC.

## Research contributions and limitations

The analysis of findings from the document analysis and interviews has revealed several factors relating to and a deeper understanding of EOC. These findings contribute to theoretical knowledge, particularly SET, and empirical knowledge, specifically with respect to Saudi Arabia. Company image, opportunity for training and development, attracting and retaining, satisfaction, involvement and commitment, fairness, work culture and environment, reward, opportunities for growth, teamwork and motivation, and concern for society emerged as the most important components of EOC in this study. Many of the researchers who have studied SET have observed that the employee–employer relationship depends on exchange, reciprocity, and a relationship that satisfies both parties. It can be considered that an EOC is also dependent on relationships and reciprocity. In the event of this association having longevity, it would be beneficial to both parties and employees would then consider the organization as an EOC.

Many employers implement practices to attract and retain talented employees. EOC involves the inculcation of holistic satisfaction, having a conducive to encouraging work culture and environment, and the overall well-being of employees. Though the majority of employees in this study held a favorable opinion about EOCs, a few lamented the lack of well-being, motivation, promotion criteria, and rigid HR practices. They considered these factors to force employees to change jobs. Management needs to consider such “flipsides” of the organization to retain talent. Researchers argue that reciprocities lead to minimization of employee turnover, maximization of commitment, satisfaction, overcoming of role stress, and creating a pleasing image of the employer in the market [28, 42].

The present study is not devoid of limitations. The first limitation of the present research relates to the external factors that might affect employees’ designation of an employer as EOC, such as cultural issues. Hence, it might be argued that the results may be unique to the Saudi context, or may not be applicable to other cultures and countries. Cultural issues can be linked to organizational culture or outside culture, depending on the country and background. For example, future studies may consider investigating the impact of employees’ cultural backgrounds on EOC. There is much room for further progress in determining how cultural factors affect EOC. As a result, further work is required to uncover new knowledge in this area.

The second limitation related to the use of a purposive sampling method to gather the information from the participants, which may influence the generalisability of the findings. However, there are multiple avenues for future research. Standardized tools and mechanisms of data analysis with different variables can be used in future research to acquire further knowledge that will spark new information assimilation about the concept of EOC in more than one organization or Small and Medium Enterprises (SMEs).

The third limitation of the present research is that the empirical result cannot be generalized because it used a single case study based on one single organization. However, the theoretical results of SET can be generalized mainly because it recognises employees who are in reciprocal interdependent relations with the employer. The results can be different from organization to organization depending on several factors, such as the type of the industry, and the size of the company.

## Data Availability

Data are available from the corresponding author upon request.

## References

[1] Ambler T, Barrow S (1996). The employer brand. J Brand Manage.

[2] Armstrong M (2006). Performance management.

[3] Aryee S, Budhwar PS, Chen ZX (2002). Trust as a mediator of the relationship between organisational justice and work outcomes: test of a social exchange model. J Organ Behav.

[4] Backhaus K, Tikoo S (2004). Conceptualizing and researching employer branding. Career Dev Int.

[5] Baldwin S (2006). Organisational justice.

[6] Blau PM (1964). Exchange and power in social life.

[7] Bowen GA (2009). Document analysis as a qualitative research method. Qual Res J.

[8] Bozionelos N, Simmering MJ (2022). Methodological threat or myth? Evaluating the current state of evidence on common method variance in human resource management research. Hum Resour Manag J.

[9] Branham L (2005). Planning to become an employer of choice. J Organ Excell.

[10] Braun V, Clarke V (2006). Using thematic analysis in psychology. Qual Res Psychol.

[11] Bryman A, Bell E (2007). Business research methods.

[12] Bryman A (2015). Social research methods.

[13] Chiaburu D, Harrison DA (2008). Do peers make the place? Conceptual synthesis and meta-analysis of lateral social influences on perceptions, attitudes, OCBs, and performance. J Appl Psychol.

[14] Clark AE, Oswald AJ (2002). A simple statistical method for measuring how life events affect happiness. Int J Epidemiol.

[15] Cropanzano R, Mitchell MS (2005). Social exchange theory: an interdisciplinary review. J Manag.

[16] Cropanzano R, Anthony E, Daniels S, Hall A (2017). Social exchange theory: a critical review with theoretical remedies. Acad Manag Ann.

[17] Farrell D, Rusbult CE (1981). Exchange variables as predictors of job satisfaction, job commitment, and turnover: the impact of rewards, costs, alternatives, and investments. Organ Behav Human Perform.

[18] Gatewood RD, Gowan MA, Lautenschlager GJ (1993). Corporate image, recruitment image, and initial job choice decisions. Acad Manag J.

[19] Guba EG, Lincoln YS (1994). Competing paradigms in qualitative research. Handb Qual Res.

[20] Herman RE, Gioia JL (2001). Helping your organization become an employer of choice. Employ Relat Today.

[21] Highhouse S, Brooks ME, Greguras G (2009). An organisational impression management perspective on the formation of corporate reputations. J Manage.

[22] Kelley HH (1968). Interpersonal accommodation. Am Psychol.

[23] Lee F, Edmondson AC, Thomke S, Worline M (2004). The mixed effects of inconsistency in organizations. Organ Sci.

[24] Lievens F, Slaughter J (2016). Employer image and employer branding: what we know and what we need to know. Annu Rev Organ Psychol Organ Behav.

[25] Lincoln YS, Guba EG (1985). Naturalistic inquiry.

[26] Macleod C. Workplace barometer, employer of choice: a reality cheque. p4. 2007. Available from www.workplacebarometer.com.au.

[27] Mau T (2019). “Enhancing leadership capacity in the public sector: branding as an employer of choice. Int J Public Leadership.

[28] Miles SJ, Mangold G (2004). A conceptualization of the employee branding process. J Relationship Market.

[29] Molm LD (1994). Dependence and risk: transforming the structure of social exchange. Soc Psychol Quart.

[30] Molm LD, Burke P (2006). The social exchange framework. Contemporary social psychological theories.

[31] Moriatry J (2010). Participation in the workplace: are employees special. J Bus Ethics.

[32] Mosley RW (2007). Customer experience, organisational culture and the employer brand. J Brand Manage.

[33] Noe RA (2010). Employee training and development.

[34] Norbit N, Nawawi A, Salin ASAP (2017). Corporate social responsibility practices among the SMEs in Malaysia—a preliminary analysis. Manage Account Rev.

[35] Rampl LV (2014). How to become an employer of choice: transforming employer brand associations into employer first-choice brands. J Mark Manage.

[36] Rupp DE, Cropanzano R (2002). The mediating effects of social exchange relationships in predicting workplace outcomes from multifoci organisational justice. Organ Behav Human Decis Process.

[37] Saini G, Jawahar I (2019). The influence of employer rankings, employment experience, and employee characteristics on employer branding as an employer of choice. Career Dev Int.

[38] Saks A (2006). Antecedents and consequences of employee engagement. J Manage Psychol.

[39] Salah MRA (2016). The impact of training and development on employees performance and productivity. Int J Manage Sci Bus Res.

[40] Saldana J (2015). The coding manual for qualitative researchers.

[41] Schneider B, Schneider B (1990). The climate for service: an application of the climate construct. Organisational climate and culture.

[42] Sharma R, Jain V, Singh SP (2018). The impact of employer branding on organisational commitment in Indian IT Sector. IOSR J Bus Manage.

[43] Silverman D (2015). Interpreting qualitative data.

[44] Sutton RI, Callahan AI (1987). The stigma of bankruptcy: spoiled organisational image and its management. Acad Manage J.

[45] Tanwar K, Kumar A (2019). Employer brand, person-organisation fit and employer of choice. Personnel Rev.

[46] Vinoth K, Vasantha S (2014). The influence of employer brand in deciding the workplace, perception of prospective employees. Int J Sci Eng Res.

[47] Ahmad A, Khan MN, Haque MA (2020). Employer branding aids in enhancing employee attraction and retention. J Asia Pacific Bus.

[48] Albinger HS, Freeman SJ (2000). Corporate social performance and attractiveness as an employer to different job seeking populations. J Bus Ethics.

[49] Allam Z (2019). Exploring ambient discriminatory HRM practices: An insight from Kingdom Telecom Company. J Soc Sci Res.

[50] Baker T. Attracting and retaining talent: becoming an employer of choice. Springer; 2014.

[51] Berg B (2007) Qualitative research methods for the social sciences. pp. 148–157

[52] Berthon P, Ewing M, Hah LL (2005). Captivating company: dimensions of attractiveness in employer branding. Int J Advertising.

[53] Bogdan R, Biklen SK (1997). Qualitative research for education.

[54] Boyatzis RE. Transforming qualitative information: Thematic analysis and code development. Sage; 1998.

[55] Cable DM, Turban DB. Establishing the dimensions, sources and value of job seekers' employer knowledge during recruitment. In: Research in personnel and human resources management. Emerald Group Publishing Limited; 2001. pp. 115-163

[56] Denzin NK. Triangulation: A case for methodological evaluation and combination. Soc Methods 1978;339–357.

[57] Ehrhart KH, Ziegert JC (2005). Why are individuals attracted to organizations?. J Manage.

[58] Francis D, Bessant J (2005). Targeting innovation and implications for capability development. Technovation.

[59] Hult GTM. Toward a theory of the boundary-spanning marketing organization and insights from 31 organization theories. J Acad Market Sci 39(4), 509–536. https://doi.org/10.1007/s11747-011-0253-6

[60] Hetrick S, Martin G (2006) Corporate reputations, branding and people management. Routledge.

[61] Jain N, Bhatt P (2015). Employment preferences of job applicants: unfolding employer branding determinants. J Manage Dev.

[62] Judge TA, Bono JE, Locke EA (2000). Personality and job satisfaction: the mediating role of job characteristics. J Appl Psychol.

[63] Karim MM, Choudhury MM, Latif WB (2019). The impact of training and development on employees’ performance: An analysis of quantitative data. Noble Int J Bus Manage Res.

[64] Knox S, Freeman C (2006). Measuring and managing employer brand image in the service industry. J Market Manage.

[65] Kucherov D, Zavyalova E (2012). HRD practices and talent management in the companies with the employer brand. Euro J Train Dev.

[66] Polanyi M, Tompa E (2004). Rethinking work-health models for the new global economy: A qualitative analysis of emerging dimensions of work. Work.

[67] Price C, Turnbull D (2007) The organizational challenges of global trends: A McKinsey global survey, McKinsey Quarterly

[68] Robertson SJ (2011) The merit of intensive leadership development programs on building-level administrators' sustainability. The University of Southern Mississippi.

[69] Rynes SL, Bretz RD, Gerhart B (1991). The importance of recruitment in job choice: A different way of looking. Pers Psychol.

[70] Sokro E (2012). Impact of employer branding on employee attraction and retention. Euro J Bus Manage.

[71] Wicks D (2017). The coding manual for qualitative researchers. Qual Res Organ Manage Int J.

